# A prognostic and therapeutic hallmark developed by the integrated profile of basement membrane and immune infiltrative landscape in lung adenocarcinoma

**DOI:** 10.3389/fimmu.2022.1058493

**Published:** 2022-11-30

**Authors:** Kaijie Chen, Shuang Liu, Changlian Lu, Xuefeng Gu

**Affiliations:** ^1^ School of Health Science and Engineering, University of Shanghai for Science and Technology, Shanghai, China; ^2^ Shanghai Key Laboratory of Molecular Imaging, Zhoupu Hospital, Shanghai University of Medicine and Health Sciences, Shanghai, China; ^3^ School of Pharmacy, Shanghai University of Medicine and Health Sciences, Shanghai, China

**Keywords:** basement membrane, immune infiltration, lung adenocarcinoma, prognosis, immunotherapy response, chemotherapy drug screening

## Abstract

Basement membranes (BMs) are specialised extracellular matrices that maintain cellular integrity and resist the breaching of carcinoma cells for metastases while regulating tumour immunity. The tumour immune microenvironment (TME) is essential for tumour growth and the response to and benefits from immunotherapy. In this study, the BM score and TME score were constructed based on the expression signatures of BM-related genes and the presence of immune cells in lung adenocarcinoma (LUAD), respectively. Subsequently, the BM-TME classifier was developed with the combination of BM score and TME score for accurate prognostic prediction. Further, Kaplan–Meier survival estimation, univariate Cox regression analysis and receiver operating characteristic curves were used to cross-validate and elucidate the prognostic prediction value of the BM-TME classifier in several cohorts. Findings from functional annotation analysis suggested that the potential molecular regulatory mechanisms of the BM-TME classifier were closely related to the cell cycle, mitosis and DNA replication pathways. Additionally, the guiding value of the treatment strategy of the BM-TME classifier for LUAD was determined. Future clinical disease management may benefit from the findings of our research.

## Introduction

Basement membranes (BMs) are thin, pliable, dense sheets of extracellular matrices (ECM) that cover the basal surface of epithelial and endothelial cells and are widely distributed around various tissues and cells in all stages of development, from embryo to adult (1, 2). The major components of BMs are collagen IV, laminin, nidogen and perlecan (3). Laminin and type IV collagen form their two-dimensional meshwork structure. Nidogen and perlecan serve as binding bridges between the two networks, assembling BM (4, 5). Diverse BMs regulate the multifaceted cellular biological process, leading to cell polarity, differentiation and migration (6–8). By acting as a physical barrier and regulating molecular exchange within and outside the cell, BM plays an important role in maintaining the integrity of cell structure and tissue separation (9). Further, BM acts as a major barrier that prevents cancer cells from breaching to develop metastases (10). Metastatic cancer is the leading cause of death in patients (11).

Lung cancer has been reported to have the highest cancer incidence and mortality rates worldwide (12, 13). Lung adenocarcinoma (LUAD) is the most prevalent histologic subtype of non-small cell lung cancer (NSCLC), accounting for approximately 40% of lung malignancies (14). According to the lung tumours chapter of the 2021 WHO Classification of Thoracic Tumours, the distinction between minimally invasive adenocarcinoma (MIA) and adenocarcinoma *in situ* (AIS) depends on whether the BM has been breached (15). With treatment or surgical resection, the five-year survival rate for AIS is 100% (16). However, once AIS penetrates the BM and develops into MIA, the prognosis will deteriorate substantially. Therefore, the BM status affects the prediction of LUAD prognosis.

Emerging evidence indicates that the tumour microenvironment (TME) contributes to the initiation and progression of cancer (17). Tumour-infiltrating immune cells in LUAD are highly heterogeneous and govern the intensity and duration of immunotherapy responses (18, 19). During tumour initiation and progression, BMs act as essential modulators in tumour immunity in addition to promoting tumour proliferation and neoangiogenesis and providing protection from chemotherapy-induced apoptosis (20). Besides, BM components also modulate diverse immune cell behaviours. Laminins not only inhibit the activation and function of CD4+ and CD8+ T cells by attenuating T cell receptor signalling but also promote their apoptosis (20). Additionally, the laminin γ2 chain regulates T cell adhesion and migration, causing T cell exclusion (21). In patients with lung tumours, increased collagen expression has been associated with elevated exhausted CD8+ T cells and the reduction of total CD8+ T cells (22). Further, collagen stabilisation correlates with tumour stiffness, thereby influencing T-cell migration (23). In essence, an intense interaction exists between BMs and tumour-infiltrating immune cells.

In this study, BM score and TME score were established based on the characteristics of BM and immune cells, respectively. According to the relationship between BM and metastasis in LUAD, further investigation of the BM score was performed for patients with and without distant metastases. Considering the interaction of the BM and immune infiltration, we developed an integrated BM-TME classifier based on the BM score and TME score for better prognosis prediction and treatment strategy guidance. Patients with LUAD in several cohorts among different subgroups exhibited diverse prognostic outcomes, enrichment pathways, somatic mutation landscape and therapeutic response, suggesting that our research findings may be conducive to the improvement of clinical disease management.

## Materials and methods

### Data source

Multiple gene expression profile datasets of LUAD samples were collected from The Cancer Genome Atlas (TCGA) database and Gene Expression Omnibus repository. Only patients with LUAD having prognostic data were retained for subsequent research. The detailed information of these cohorts was summarised in **Table S1**. In this study, the TCGA-LUAD cohort was applied as the training set for constructing the BM score and TME score. Meanwhile, independent validation sets consisted of five datasets [GSE30219 (24), GSE50081 (25), GSE37745 (26), GSE81089 (27) and GSE135222 (28)], including microarray and RNA sequencing data. Additionally, single-cell RNA sequencing data from eight primary and five metastatic LUAD tumours were retrieved from GSE123902 (29) to visualize BM scores in each cell. Gene levels with survival data for 32 cancers were retrieved from UCSC Xena (https://xena.ucsc.edu/) (30). The bulk RNA sequencing data were log2 (TPM + 1) transformed for further analysis.

### Screening prognostic BM-related genes and immune cells for establishing BM score and TME score

The set of 160 BM network genes was derived from a recently published paper (31). To identify prognostic-related BM genes in LUAD, we performed differential expressed gene (DEG) analysis, univariate Cox regression analysis and least absolute shrinkage and selection operator (lasso) regression analysis of 160 BM genes in TCGA-LUAD cohort successively. The threshold of DEGs was set as p.adjust < 0.05. CIBERSORT is an algorithm for estimating 22 immune cells composition of various tissue from gene expression signatures and is used in TCGA-LUAD cohort (32). Subsequently, Kaplan–Meier survival curve estimation was executed to identify the prognostic value of immune cells. Immune cell types with favorable prognosis and p-value of Kaplan-Meier analysis less than 0.01 were selected for constructing the TME model.

### Development of BM score, TME score and BM-TME classifier

The coefficients (Coef) for multivariate Cox regression analysis of 20 BM genes and three types of immune cells in TCGA-LUAD cohort were the basis of the establishment of BM score and TME score. To improve the accuracy of both BM and TME models, we conducted 1000 random sampling of all LUAD samples and performed multivariate Cox analyses each time. Furthermore, the standard deviation (SD) values of Coef were acquired for each gene and cell. Their weights in the corresponding models depend on the ratio of the Coef to the SD values. In summary, the BM score was given as the following formula:


$$BM\;score=\Sigma_{i=1}^{20}\frac{Coef_{i}}{SD_{i}}*exp(gene_{j}).$$

Similarly, the formula for the TME score was as follows:


$$TME\;score=\Sigma_{j=1}^{3}\frac{-Coef_{j}}{SD_{j}}*fra(cell_{j}).$$

Where *exp*(*gene_i_*) and *fra*(*cell_j_*) indicate the expression level of gene i and the fraction of cell j, respectively. Thereafter, the BM-TME classifier was developed based on the median value of the BM and TME score of each data set. The samples of each cohort were classified into “BM_low+TME_high”, “Mixed” (BM_low+TME_low, BM_high+TME_high) and “BM_high+TME_low” groups. Depiction of receiver operating characteristic (ROC) curves was utilised to measure the prognostic predictive ability of the BM-TME classifier through the “timeROC” package (33).

### Visualization of BM score at the single cell level

To perform the clustering analysis, annotation and visualization for the single cell data, we created Seurat objects for scRNA-seq gene expression matrix (34). Transcriptomes with more than 300 and fewer than 6000 expressed genes were remained. Cells with more than 50000 reads or mitochondrial genes occupying more than 15% reads were filtered out. Then, the top 3000 highly variable genes were selected for reducing the dimensionality using principal component (PC) analysis. Further, t-SNE (t-distributed stochastic neighbor embedding) was used to summarize the top 30 PC and visualize the single cell data. We employed FindNeighbors and FindClusters functions to identify distinct cell clusters. Cell type annotations were determined based on canonical cell type markers collected from Bischoff et al (**Table S2**) (35). The BM score for each cell was calculated according to the above formula of BM model.

### Gene set functional annotation and enrichment analysis

Gene set enrichment analysis (GSEA) was performed to explore the Kyoto Encyclopedia of Genes and Genomes (KEGG) pathways potentially associated with BM score and TME score. Weighted gene co-expression network analysis (WGCNA) was applied for the scout of the gene module affecting the BM-TME classifier (36). The genes with the top 5000 median absolute deviation in TCGA-LUAD expression profile were retrieved for WGCNA analysis. Their biological functions were subsequently discovered *via* gene ontology (GO) analysis. The above enrichment analyses were implemented using the “clusterProfiler” package (37). Additionally, proteomaps were generated by importing a list of differentially expressed proteins on an online tool (https://proteomaps.net/) (38).

### Somatic mutation and immunotherapy response

The mutation annotation format (MAF) data of TCGA-LUAD cohort was accessible in TCGA database. The “maftools” package was used to create waterfall plots of the top 15 mutated genes to compare the somatic mutation status in different groups of BM-TME classifiers (39). Additionally, the tumour mutational burden (TMB) of each LUAD sample was obtained by calculating the total number of somatic mutations per million bases in the tumour genome following the removal of germ-line mutation. Tumour immune dysfunction and exclusion (TIDE) database infers the function of genes that regulate tumour immunity and predict the response of anti-programmed cell death 1 (anti-PD1) and anti-cytotoxic T-lymphocyte associated protein 4 (anti-CTLA4) for melanoma and NSCLC (40). Genomics of Drug Sensitivity in Cancer (GDSC) database collected two datasets on the sensitivity and response of tumour cells to drugs: GDSC1 and GDSC2 (41). Taking the LUAD cells in the GDSC2 cohort as the training set, the half-maximal inhibitory concentration (IC50) for various chemotherapeutic drugs of patients with LUAD in TCGA-LUAD and GSE30219 cohort was predicted with the “oncoPredict” package (42).

### Statistical analysis

All the statistical analysis of this article was completed on R 4.1.0. Correlations between variables were analysed using Pearson and Spearman methods. Comparisons between different subgroup samples were performed using nonparametric tests, including the Wilcoxon and Kruskal–Wallis rank sum tests. A *p*-value of <0.05 was valuable. “*”, “**” and “***” indicated *p*<=0.05, *p*<=0.01 and *p*<=0.001, respectively.

## Results

### Both BM score and TME score are prognostic valuable but have the opposite effect

**Figure 1:**
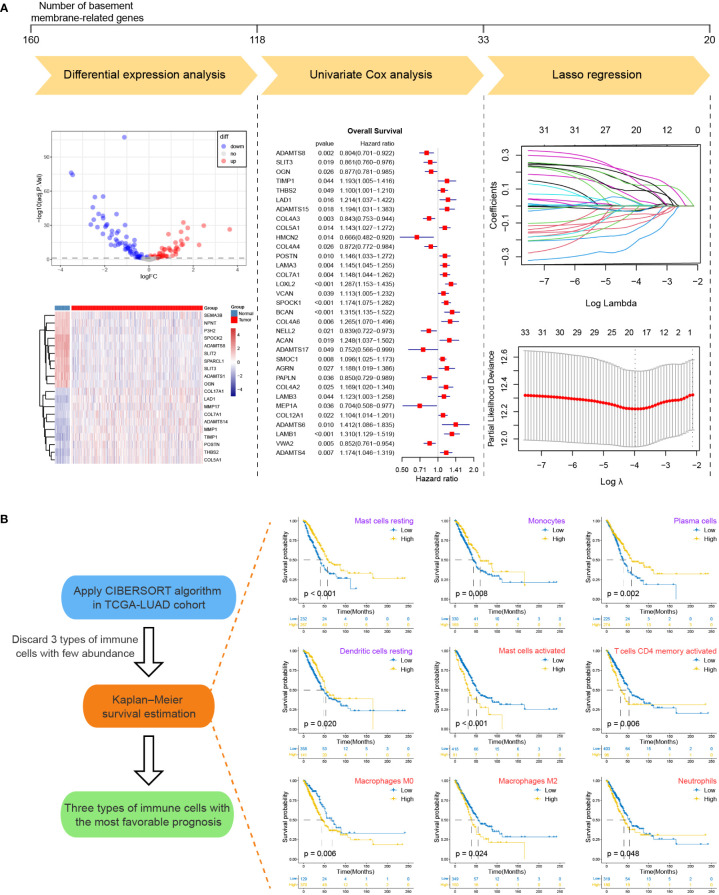
The workflow for screening basement membrane-related genes and immune cell types for establishing the BM score and TME score. **(A)** Differential expression analysis, univariate Cox regression analysis and lasso regression analysis were performed for 160 BM-related genes in TCGA-LUAD cohort in succession. The upper line marked the number of BM-related genes before and after each step analysis. Briefly, 20 BM-related genes were selected for the development of BM score. **(B)** The CIBERSORT algorithm was applied to generate the abundance of 22 types of immune cells for TCGA-LUAD samples. After abandoning 3 types of immune cells with few abundances, Kaplan-Meier survival curve estimation was executed for the remaining immune cell types. Kaplan-Meier overall survival curves were shown on the right side only for immune cell types with p-values less than 0.05 (purple indicated favorable prognostic factor while red represented unfavorable prognostic factor). Ultimately, mast cells resting, monocytes and plasma cells were used for the establishment of TME score.

**Figure 2:**
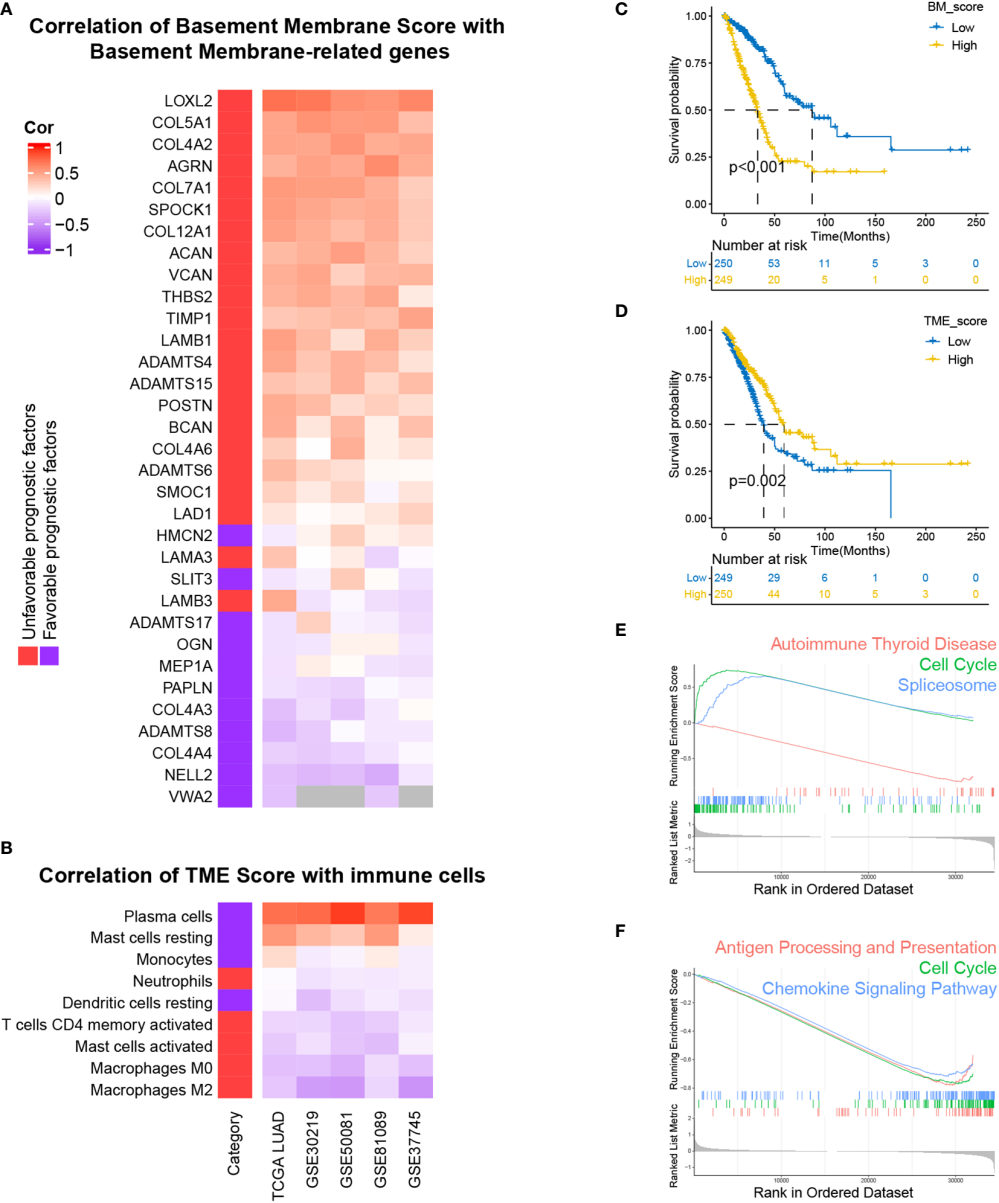
Development and performance of BM and TME scores in LUAD, respectively. **(A, B)** The correlation of BM score and TME score with the expression levels of prognostically BM genes and the abundance of prognostically immune cells. **(C, D)** Kaplan–Meier survival curves of high and low BM, TME scores subgroups. **(E, F)** Top three KEGG enrichment pathways based on GSEA analysis of differentially expressed genes (DEGs) between high and low BM, TME scores groups.

For the sake of developing a method to estimate the basement membrane and immune cell status of patients with LUAD, DEG analysis, univariate cox analysis and lasso regression analysis of BM genes and Kaplan-Meier overall survival estimation of immune cells were conducted sequentially in TCGA-LUAD dataset (**Figures 1A, B**). The details of the workflow for screening 160 BM genes and 22 immune cells were presented in **Tables S3**, **4**. Afterward, BM score and TME score were established with 20 BM genes and three types of immune cells, respectively. Their information was in the **Table S5**. The heatmaps in **Figures 2A, B** separately demonstrated the association of BM score and TME score with prognostic-related BM genes and immune cells in five different LUAD cohorts. According to the overall outcome, BM scores were positively correlated with the majority of unfavourable prognostic factors and negatively correlated with favourable prognostic factors. In contrast, the TME score primarily had positive relationships with favourable prognostic immune cells. Besides, the results of Kaplan–Meier overall survival curves revealed that patients with LUAD in the low BM score group had a better survival outcome than those in the high BM score group, however, the situation with TME score was reversed (**Figures 2C, D**). These results were hardly surprising because the TME score was constructed with negative Coef while the BM score was not. To identify the potential differential KEGG pathways between high and low BM and TME groups, the top three pathways for the results of GSEA were displayed (**Figures 2E, F**). Both were enriched in the cell cycle pathway. Additionally, pathways associated with BM score included DNA replication and ECM receptor interaction (**Table S6**). The differentially expressed genes (DEGs) between high and low TME score tumours were enriched in immune-related pathways, such as the T cell receptor and the JAK-STAT signalling pathways (**Table S7**).

### BM score is diminished in immune cells and elevated in tumours with metastatic

**Figure 3:**
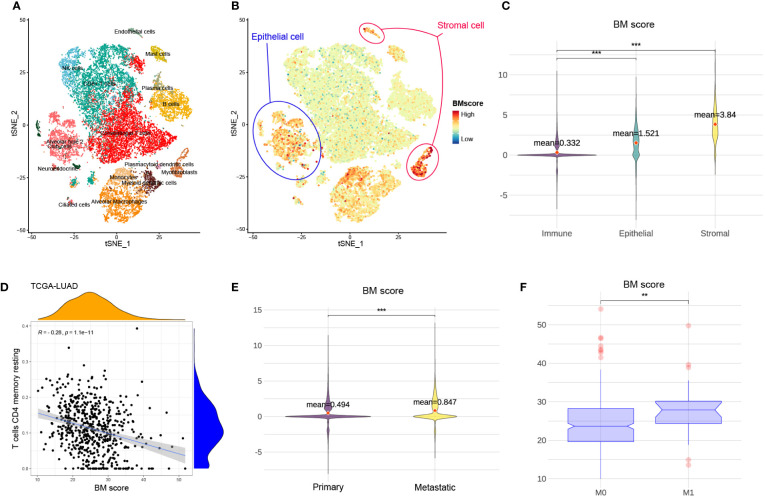
The relationship between BM score and different types of cells and tumours. **(A)** t-SNE scatters plot of eight primary and five metastatic LUAD samples with cell annotation. **(B)** The distribution of BM scores at the single cell level. Blue and red circles represent epithelial and stromal cells, respectively. The rest of the scatter plot is filled with immune cells. **(C)** The violin plot demonstrating the difference in BM score among immune, stromal and epithelial cells. And the red dots indicate the average value of BM scores for each group. **(D)** The relationship between BM score and the abundance of T cells CD4 memory resting in TCGA-LUAD cohort. **(E)** The violin plot showing the difference in BM score between primary and metastatic LUAD tumour cells. Further, the red dots indicate the average value of BM scores for each group. **(F)** The distribution of BM score between tumours with non-metastatic (M0) and metastatic (M1). **p<=0.01 and ***p<=0.001, respectively.

To visualize BM score at cell levels and explore potential associations of BM score among multiple types of tissue and cells, t-SNE plots of 23899 cells from eight primary and five metastatic LUAD biospecimens were generated (**Figures 3A, B**). The main cell types were defined based on the canonical markers for distinct cell types (**Figure S1**). When compared to stromal or epithelial cells, immune cells had a significantly lower BM score (**Figure 3C**). Moreover, there was a negative correlation between BM score and the abundance of resting CD4 memory T cells among several cohorts (**Figure 3D**, **S2**). These findings suggest that low BM scores in patients with LUAD may be associated with increased immune cell proportions especially resting CD4 memory T cells. Similarly, compared to primary tumours (M0), patients with LUAD who had metastatic tumours (M1) had a significantly increased BM score (**Figures 3E, F**). Therefore, a high BM score was a red flag of tumour metastasis in patients with LUAD.

### BM-TME classifier was an independent prognostic indicator for multipleLUAD subtypes

**Figure 4:**
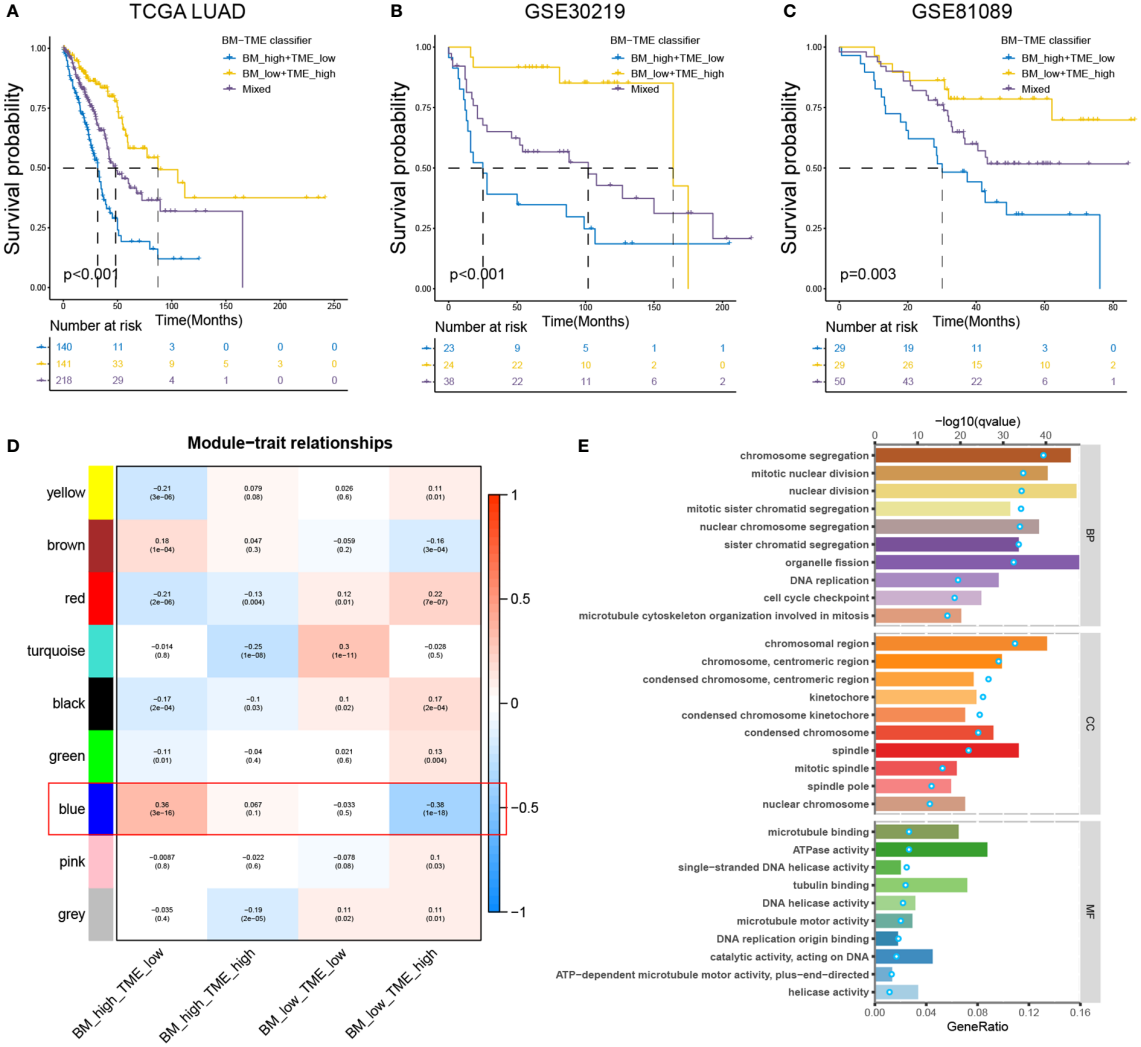
Prognostic value and enrichment analysis relevant to BM-TME classifier. **(A–C)** Kaplan–Meier overall survival curves of the training set (TCGA-LUAD cohort) and validation sets (GSE30219 and GSE81089 cohort) based on BM-TME classifier. **(D)** Heat map depicting the correlation between various gene modules of WGCNA analysis and BM-TME subgroups. **(E)** Top ten biological process (BP), cellular component (CC) and molecular function (MF) enrichment pathways based on GO analysis of blue module genes. Bar plots correspond to the lower axis; dot plots correspond to the upper axis.

According to the aforementioned findings, we considered if it could be preferable to combine the BM score and TME score to simultaneously characterise the BM and immune microenvironmental status of the tumour. The BM-TME classifier was then constructed based on the median value of BM score and TME score for each cohort. Kaplan–Meier overall survival curves were used to analyse the prognostic predictive capability of the BM-TME classifier. In TCGA-LUAD cohort, patients in the “BM_low+TME_high” subgroup had the best prognostic outcomes, followed by the “Mixed” subgroup, and the “BM_high+TME_low” subgroup had the worst prognosis (**Figure 4A**). The same analysis results were observed in GSE30219 and GSE81089 datasets (**Figures 4B, C**).

WGCNA analysis was performed to scout for the gene module related to the BM-TME classifier (for more details, see **Figure S3** and **Table S8**). As illustrated in **Figure 4D**, the blue module genes were most strongly correlated with the BM-TME subgroups. Subsequently, GO analysis was conducted for these genes (**Figure 4E**). Overall, the enrichment analysis results revolve around the biological processes of the cell cycle, such as DNA replication, mitotic spindle, sister chromatid segregation and mitotic nuclear division. It was interesting to discover the combined analysis of BM and TME synergistically correlates to cancer cell proliferation.

**Figure 5:**
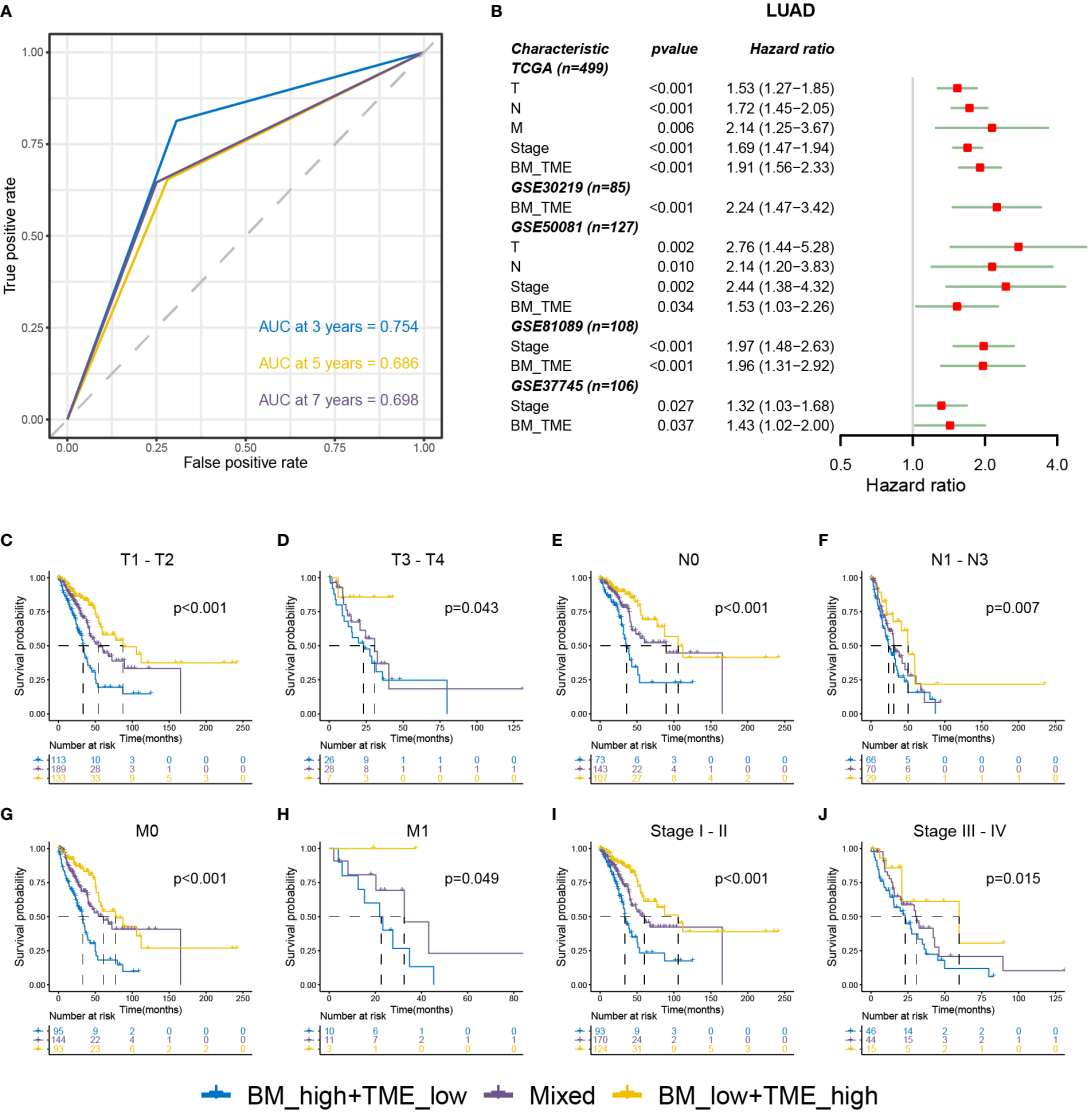
Relationships between BM-TME classifier and clinical features in LUAD. **(A)** ROC curves for the 3-, 5- and 7-year overall survival based on the BM-TME classifier in TCGA-LUAD cohort. **(B)** Univariate Cox analysis of clinical characteristics and BM-TME classifier in five LUAD cohorts. **(C–J)** Kaplan–Meier overall survival curves of BM-TME classifier in diverse LUAD clinical subtypes in TCGA-LUAD cohort.

Receiver operating characteristic (ROC) analysis indicated that the BM-TME classifier can predict overall survival at 3, 5 and 7 years with the area under the curve of 0.754, 0.686 and 0.698, respectively (**Figure 5A**). Univariate Cox analysis of five LUAD cohorts revealed that the TNM, stage and BM-TME classifier were all unfavourable prognostic factors (**Figure 5B**). In addition, the prognosis predictive performance of the BM-TME classifier was comparable to the stage. To investigate and extend the generalised predictive ability of the BM-TME classifier in tumours, a univariate Cox analysis of 32 cancers suggested that the BM-TME classifier was also an unfavourable prognostic indicator for six cancers, including adenoid cystic carcinoma, mesothelioma, low-grade gliomas, sarcoma, cervical squamous cell carcinoma and head and neck squamous cell carcinoma (**Figure S4**). Furthermore, Kaplan–Meier overall survival curves of BM-TME classifier in multiple LUAD clinical subtypes were performed (**Figures 5C–J**). The results revealed that the BM-TME classifier performed effectively in the prognostic prediction of different TNM and stage subtypes of LUAD, which may contribute to validating the general applicability of the BM-TME classifier in predicting prognosis in LUAD.

### Different somatic mutation landscapes among BM-TME subgroups

**Figure 6:**
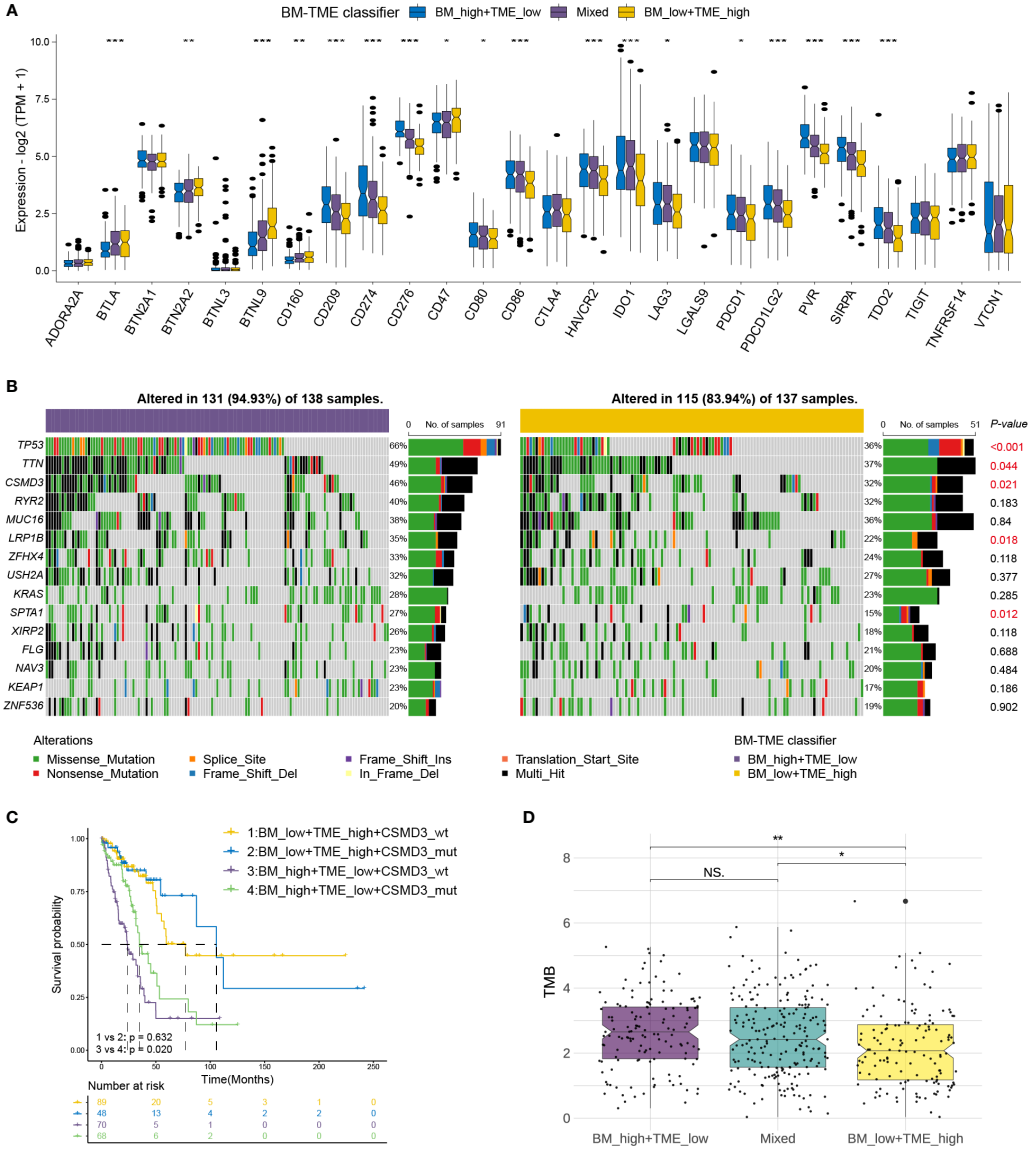
Correlations of immune checkpoints and somatic mutation with BM-TME classifier in TCGA-LUAD cohort. **(A)** The differential expression levels of immune checkpoint genes among BM-TME classifier subgroups. **(B)** Waterfall plots depicting the mutation landscape of the top 15 genes with high mutation frequency. *P*-values on the right side displaying the significance of differences in 15 gene mutation frequencies between two BM-TME groups. **(C)** Kaplan–Meier curves of patients with LUAD divided by the *CSMD3* mutation status and BM-TME classifier. **(D)** The distribution of TMB among different BM-TME classifier subgroups. *p<=0.05, **p<=0.01 and ***p<=0.001, respectively. ns, no significance.

Immune checkpoint therapy provides lasting clinical benefits to oncology patients (43). Thus, the expression patterns of prominent checkpoint genes were further investigated among different BM-TME subgroups. Differential expression was observed among most checkpoint genes in TCGA-LUAD (18/27) and GSE50081 (21/27) cohorts (**Figure 6A**, **S5**). Furthermore, the expression of 13 checkpoint genes was downregulated in the “BM_low+TME_high” group compared to those in the “Mixed” and “BM_high+TME_low” groups, such as IDO1, CD274, PDCD1, HAVCR2, and so on. In addition, the “BM_low+TME_high” subgroup exhibited elevated mRNA levels of CD160, BTLA, BTN2A2, BTNL9 and CD47 than the other two subgroups.

Somatic mutations occur and accumulate throughout a person’s life. One theory suggests that cancer occurs and develops owing to genetic mutations that accumulate over time (44). We further identified the somatic mutation landscape among different BM-TME subgroups. The top 15 genes in mutation frequency in “BM_low+TME_high” and “BM_high+TME_low” groups are demonstrated with waterfall diagram in **Figure 6B**. Compared to the “BM_high+TME_low” group, the “BM_low+TME_high” group had a lower frequency of both gene mutations and mutations occurring in patients. The mutation frequencies of *tumour protein P53*, *titin*, *cub and sushi multiple domain 3* (*CSMD3*), *low-density lipoprotein receptor-related protein 1B* and SPTA1 were significantly different between these two groups. Besides, “BM_high+TME_low” subgroup patients who had *CSMD3* wildtype may have the worse prognosis (**Figure 6C**). The “BM_low+TME_high” group had the lowest TMB compared to the other two groups, which corresponded to the results for mutation frequency (**Figure 6D**).

### BM-TME classifier-guided LUAD treatment strategies

**Figure 7:**
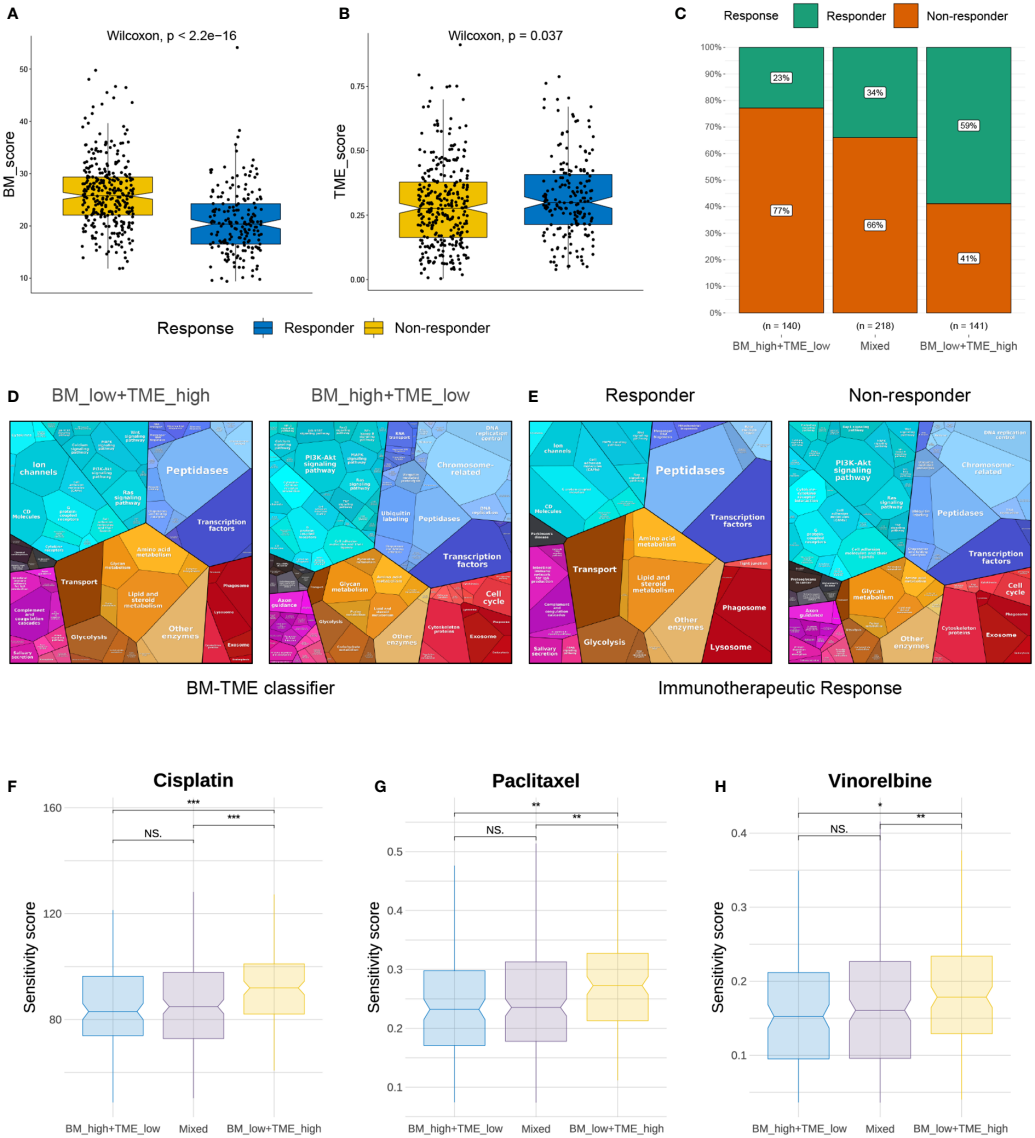
Immunotherapy response and chemotherapy drug screening prediction. **(A, B)** Differential distribution of BM score and TME score in immunotherapy response and non-response groups. **(C)** Comparison of immunotherapy responses among different BM-TME classifier groups in TCGA-LUAD cohort. **(D, E)** Proteomaps of the functional analysis results in patients of “BM_low+TME_high”, “BM_high+TME_low”, immunotherapy responder and non-responder groups. Each KEGG pathway is represented by a polygon, and the size of polygons corresponds to the protein ratio. **(F–H)** Comparison of drug sensitivity to cisplatin, paclitaxel and vinorelbine among different BM-TME subgroups. *p<=0.05, **p<=0.01 and ***p<=0.001, respectively. ns, no significance.

Immunotherapy and chemotherapy are currently the most common strategies for cancer treatment. The TIDE module can effectively predict the response to anti-PD1 and anti-CTLA4 therapy in patients with NSCLC (45). We next used TIDE to predict the response of different BM-TME groups to immunotherapy. A diminished BM score and elevated TME score were observed in the responder group compared to those in the non-responder group (**Figures 7A, B**). Correspondingly, 59%, 34% and 23% of patients in the “BM_low+TME_high”, “Mixed” and “BM_high+TME_low” subgroups responded to immunotherapy, respectively (**Figure 7C**). Additionally, the similar results were discovered in the GSE30219 cohort and another clinical immunotherapy cohort (GSE135222) treated with anti-PD-1/PD-L1 (**Figure S6**). It was evident that patients in the “BM_low+TME_high” subgroup were more likely to benefit from immunotherapy than those in the other two subgroups. Besides, the proteomaps were used to visually demonstrate and contradistinguish the underlying mechanisms among patients with LUAD in different groups (**Figures 7D, E**). Interestingly, the proteomaps of “BM_low+TME_high” and responder groups exhibited a considerably high degree of similarity. A similar result was observed between the “BM_high+TME_low” and non-responder groups. This suggests that the BM-TME classifier can effectively reflect the TME of patients with LUAD and predict the outcome of immunotherapy.

In contrast, sensitivity scores were yielded to predict the IC50 of chemotherapeutic agents using the “oncoPredict” package. As presented in **Figures 7F–H**, cisplatin, paclitaxel and vinorelbine may be more effective options for “BM_high+TME_low” and “Mixed” subgroups than for the “BM_low+TME_high” subgroup. However, the sensitivity score for cisplatin, which targets the DNA replication pathway, was much higher than those for paclitaxel and vincristine, which target mitosis. In addition, DNA replication and mitotic pathways were enriched in the previous GO function analysis (**Figure 4E**). These results suggest that chemotherapeutic agents targeting mitosis may be an effective strategy for treating LUAD.

## Discussion

BM, a specialised ECM that maintains cell compartmentation and structural integrity, is the predominant barrier that carcinoma cells must consistently breach to form metastases (10, 46). In addition, by weakening T cell activation, BM and its components facilitate tumour progression (20). Based on 20 screened BM genes, the BM score was constructed for prognostic prediction in LUAD. Interestingly, BM score exhibited positive and negative correlations with unfavourable and favourable prognostic factors, respectively, for the 33 prognosis-associated BM genes. Further, elevated BM scores in patients with LUAD implied a poor prognosis, weakened immune cells and an increased risk of developing metastases, revealing that the BM score and its 20 BM genes may be used to describe the status and characteristics of BM in LUAD tumour tissue. Afterwards, the TME score was built based on the presence of the immune cells. GSEA analysis revealed that the cell cycle, the pathway affecting the prognosis of patients with LUAD (47), were associated with both the BM score and TME score, suggesting its important role in the development of LUAD.

Considering the strong interaction between BMs and immune cells, an integrated classifier was established by combining BM score and TME score for comprehensive and accurate prognosis prediction. In six other cancer cohorts and several LUAD cohorts, patients of the “BM_low+TME_high” subgroup exhibited better survival outcomes than those in the other two BM-TME subgroups, demonstrating the universal applicability of the BM-TME classifier in patients with carcinoma. This also implied that patients with various types of cancer shared certain characteristics related to BM and immune infiltration.

GO function enrichment analysis of the gene module that was most relevant to the BM-TME classifier elucidated that its underlying molecular mechanisms predicting prognosis and classification were primarily associated with mitosis and DNA replication processes. LY6K-AS lncRNA and maternal embryonic leucine zipper kinase act as oncogenic molecules by regulating the mitotic process of LUAD cells (48, 49). DEAD-box helicase 59 plays an important role in LUAD development by promoting DNA replication (50). Intriguingly, the commonly used chemotherapeutic agents, vinorelbine, paclitaxel and cisplatin for patients with LUAD target the mitotic and DNA replication pathways, respectively. A study by Gonzalez et al. revealed that a cell cycle-dependent cisplatin-resistant mechanism was associated with mitosis and DNA replication process (51). The aforementioned results revealed that mitosis and DNA replication may serve as promising therapeutic target pathways for LUAD.

The research on somatic mutational signatures with different BM-TME groups remarkably discovered that the expression profile-based BM-TME classifier also reflects DNA heterogeneity. A combination of *CSMD3* mutation status and the BM-TME classifier may cause a better survival prediction. A poor survival outcome was associated with *CSMD3* wildtype, and the same result was discovered in lung squamous cell carcinoma (52). In general, the frequency of mutations in multiple genes was higher in the “BM_high+TME_low” subgroup than in the “BM_low+TME_high” subgroup. Furthermore, higher TMB was observed in patients of the “BM_high+TME_low” subgroup than in those of the “BM_low+TME_high” subgroup. Immune checkpoint disruption can activate the body’s natural anti-tumour defence (53, 54), and distinct BM-TME subgroups displayed diverse immune checkpoint expression patterns, indicating that each group may have responded differently to immunotherapy. In addition, 59% of patients in the “BM_low+TME_high” subgroup, 23% of those in the “BM_high+TME_low” subgroup and 34% of those in the “Mixed” subgroup responded to immunotherapy. The similarity between the proteomaps of the “BM_low+TME_high” and immunotherapy response groups reveals certain immune system commonalities between the two patient groups, further demonstrating the efficacy of the BM-TME classifier for directing therapeutic strategies in LUAD.

Overall, we identified the BM score and TME score separately and combined them to establish the BM-TME classifier for LUAD prognostic prediction and treatment strategy guidance. It might be a potential approach for future prognosis estimates and patient stratification for clinical disease management. However, our study had some limitations. First, although the survival prediction value of the BM-TME classifier was validated using several publicly available datasets, there was a dearth of in-house data to fully evaluate its functionality. Second, the classification of patients by the BM-TME classifier was based on the median values of the BM score and TME score for a group of patients. Therefore, we were unable to accurately classify the condition when there was just one patient with LUAD. Similar to the challenges encountered with the definition of the h-TMB cut-offs (55), a significant amount of standardised calibration data is needed for uniform definition in the future.

## Data availability statement

The datasets presented in this study can be found in online repositories. The names of the repository/repositories and accession number(s) can be found in the article.

## Author contributions

XG and CL conceived and designed the study. KC and SL were responsible for the collection and assembly of data, data analysis, interpretation, and the writing of the manuscript. XG and CL provided help in revising the manuscript. Our team is Innovative team of intelligent inspection and active health (ITIH). All authors contributed to the article and approved the submitted version.

## Funding

This work was supported by the National Natural Science Foundation of China (81772829 and 81830052), the Special Program for Collaborative Innovation, the Construction project of Shanghai Key Laboratory of Molecular Imaging (18DZ2260400) and “Top-100 Talent Cultivation Plan” of Shanghai University of Medicine and Health Sciences, and Funding Scheme for Training Young Teachers in Shanghai Colleges.

## Acknowledgments

We thank Bullet Edits Limited for the linguistic editing and proofreading of the manuscript.

## Conflict of interest

The authors declare that the research was conducted in the absence of any commercial or financial relationships that could be construed as a potential conflict of interest.

## Publisher’s note

All claims expressed in this article are solely those of the authors and do not necessarily represent those of their affiliated organizations, or those of the publisher, the editors and the reviewers. Any product that may be evaluated in this article, or claim that may be made by its manufacturer, is not guaranteed or endorsed by the publisher.
