# Supplementary material for: A prognostic and therapeutic hallmark developed by the integrated profile of basement membrane and immune infiltrative landscape in lung adenocarcinoma
Source: Front Immunol. 2022 Nov 30;13:1058493. doi: 10.3389/fimmu.2022.1058493 (PMC9748099; doi:10.3389/fimmu.2022.1058493)

Figure S1. Dotplot of 12 markers representative of each cell type.

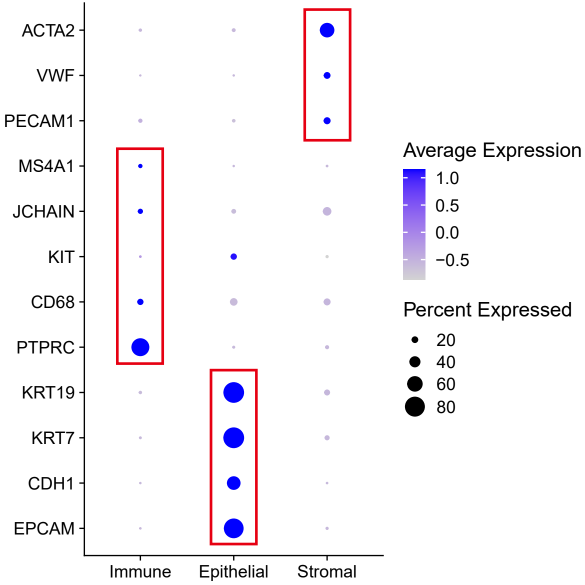

Figure S2. The relationship between BM score and the abundance of T cells CD4 memory resting in GSE30219 and GSE81089 cohorts.

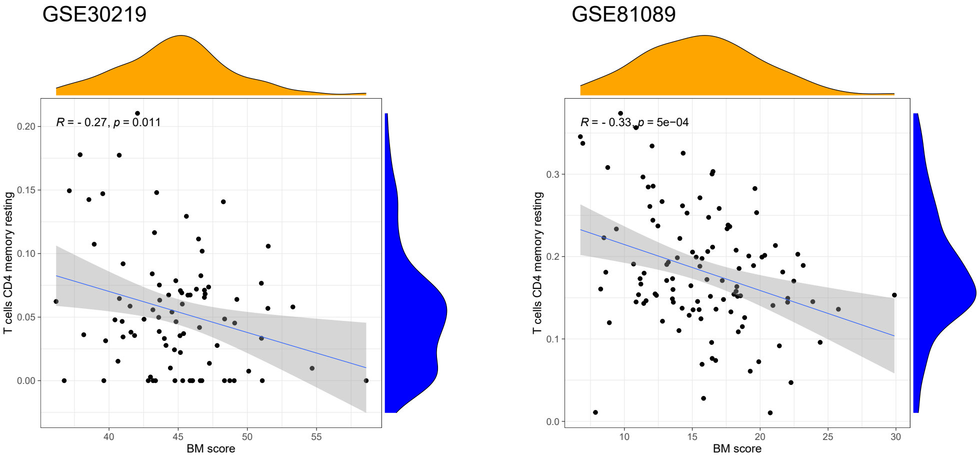

Figure S3. WGCNA analysis. (A) Clustering dendrogram of TCGA-LUAD cohort before sample filtering. (B) Clustering dendrogram of TCGA-LUAD cohort after sample filtering. (C) Analysis of the scale-free index and mean connectivity for various soft-threshold powers (β). (D) WGCNA cluster dendrogram and module assignment. The branches refer to clusters of genes that are highly connected. The colors in the horizontal bar represent the modules.

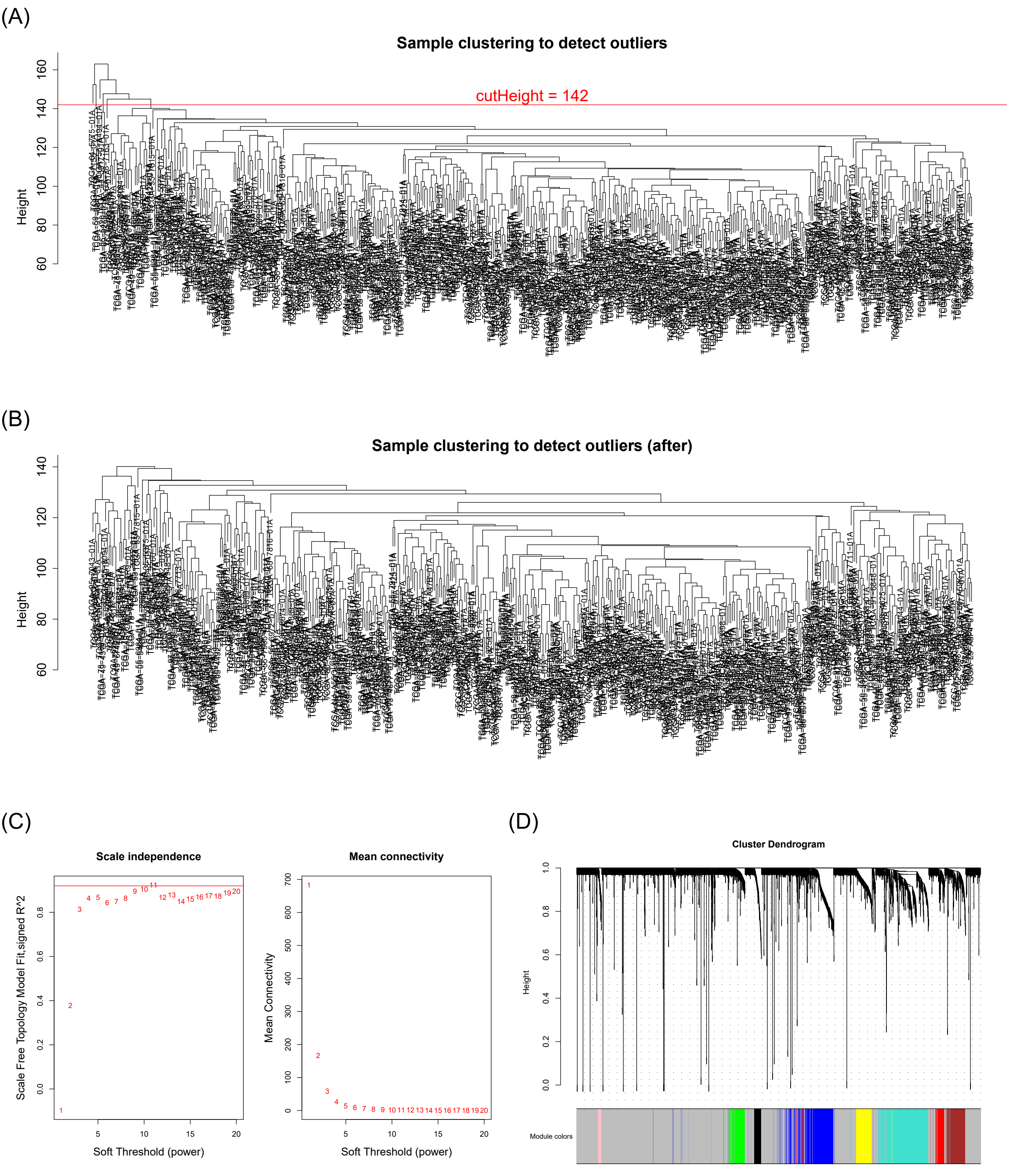

Figure S4. Evaluation of the prognostic value of BM-TME classifier in 32 pan-cancer cohorts.

BM−TME classifier

Cancer	Number	P value	Hazard ratio
TGCT	138	0.207	2.92(0.55−15.45)
ACC	79	0.006	2.12(1.24−3.62)
THCA	504	0.070	2.01(0.94−4.27)
MESO	79	<0.001	1.92(1.38−2.67)
UVM	77	0.093	1.68(0.92−3.07)
LGG	509	<0.001	1.50(1.19−1.89)
SARC	259	0.009	1.44(1.10−1.90)
CESC	285	0.019	1.43(1.06−1.94)
HNSC	496	0.007	1.27(1.07−1.52)
PAAD	177	0.190	1.20(0.91−1.58)
THYM	118	0.755	1.20(0.38−3.74)
UCS	54	0.456	1.20(0.74−1.96)
LUSC	489	0.107	1.17(0.97−1.41)
BLCA	400	0.196	1.15(0.93−1.42)
KIRC	523	0.231	1.15(0.91−1.45)
LIHC	365	0.301	1.13(0.90−1.42)
BRCA	1057	0.363	1.11(0.89−1.38)
KIRP	285	0.692	1.09(0.72−1.65)
LAML	117	0.602	1.09(0.80−1.48)
SKCM	455	0.365	1.09(0.91−1.31)
COAD	432	0.588	1.08(0.82−1.42)
GBM	155	0.511	1.07(0.87−1.33)
CHOL	36	0.866	1.06(0.54−2.10)
READ	155	0.866	1.04(0.63−1.74)
OV	358	0.846	1.02(0.84−1.24)
STAD	348	0.888	1.02(0.81−1.27)
UCEC	534	0.904	0.98(0.74−1.31)
PRAD	482	0.909	0.95(0.41−2.20)
DLBC	46	0.901	0.94(0.34−2.59)
ESCA	152	0.718	0.94(0.67−1.32)
PCPG	180	0.503	0.72(0.28−1.88)
KICH	64	0.396	0.65(0.24−1.75)

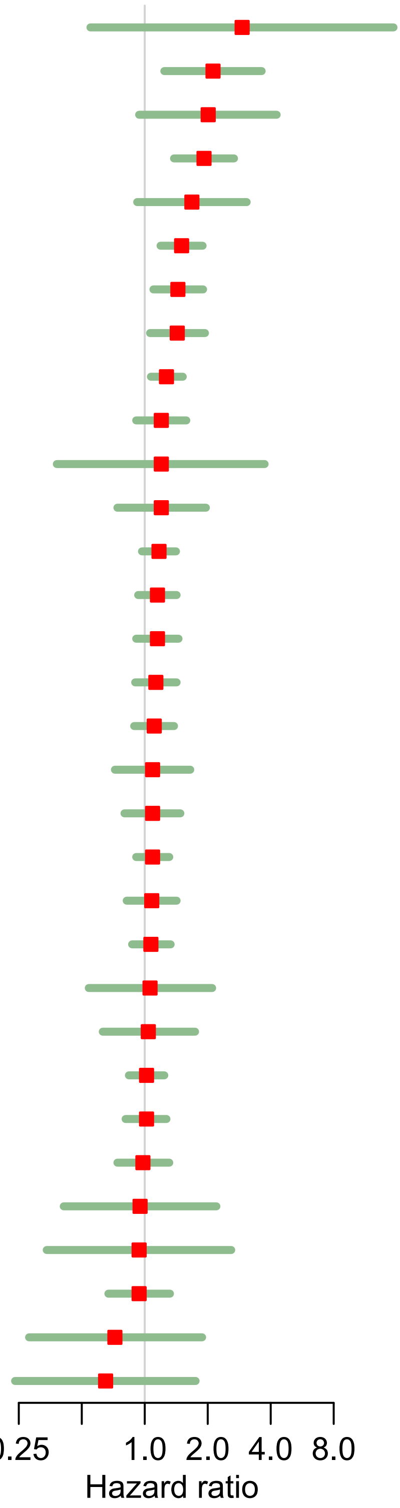

Figure S5. The differential expression levels of immune checkpoint genes among BM-TME classifier subgroups in GSE50081 cohort.

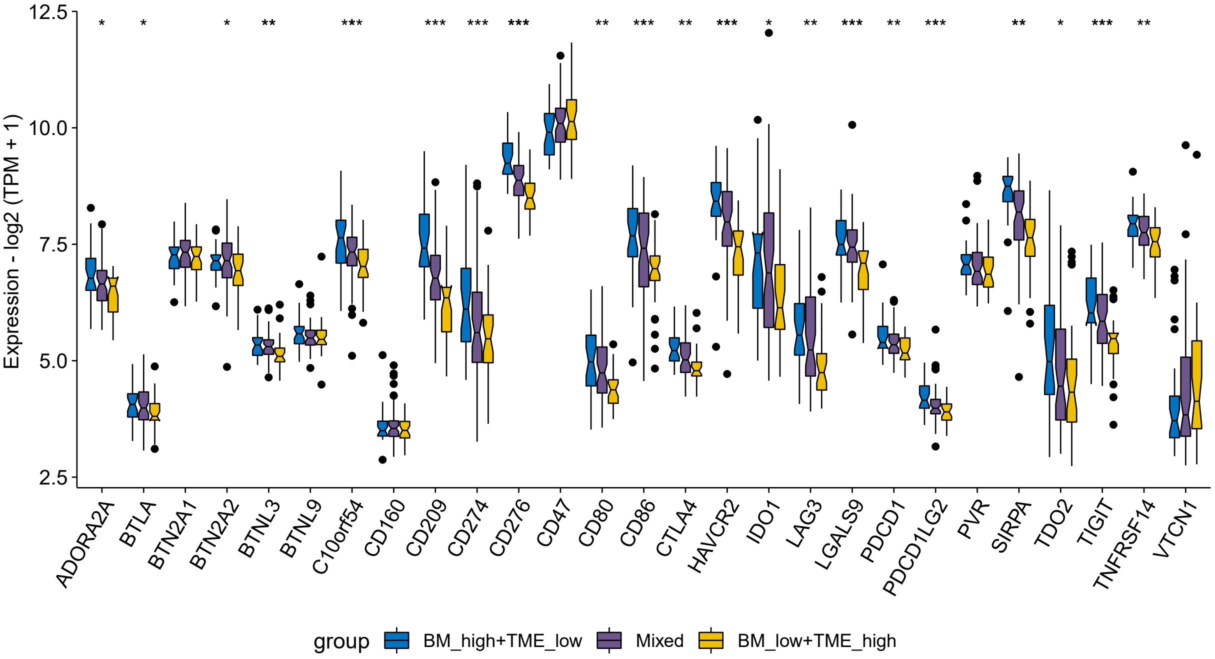

Figure S6. Comparison of immunotherapy responses among different BM-TME classifier groups in GSE30219 and GSE135222 cohorts.

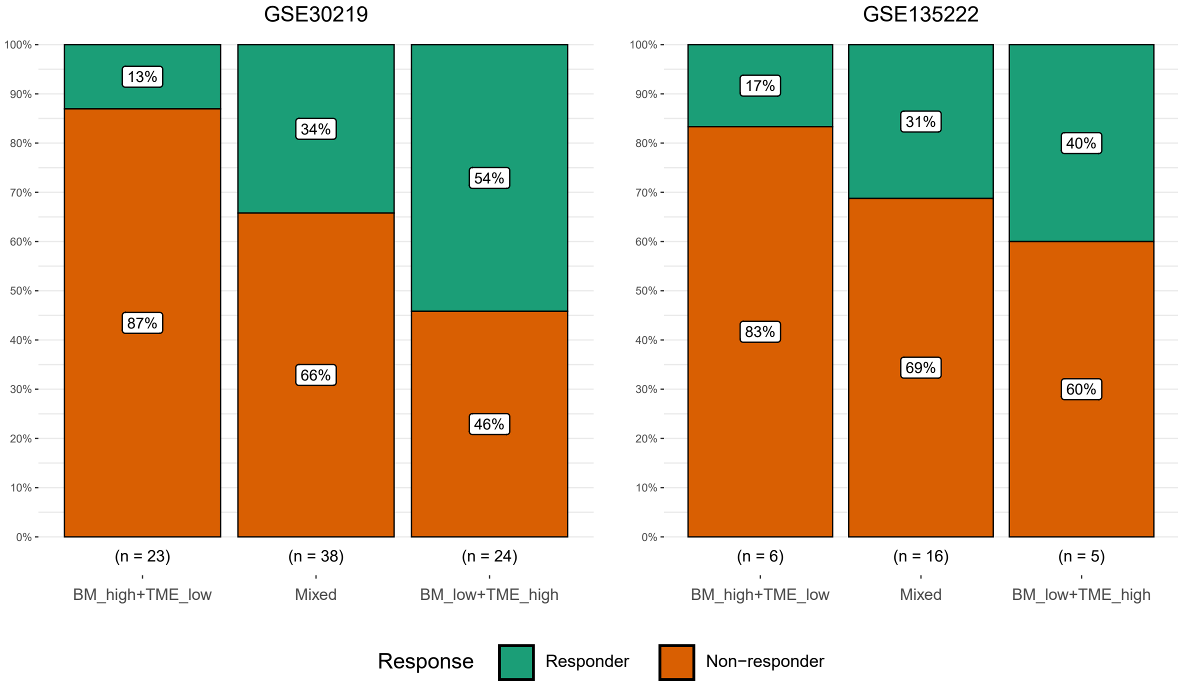

Supplement: Supplementary file 1 [file Image_1.pdf]
